# Thermal Stability of Polydivinylbenzene and of Copolymers of Styrene With Divinylbenzene and With Trivinylbenzene^1^

**DOI:** 10.6028/jres.065A.029

**Published:** 1961-06-01

**Authors:** Sidney Straus, Samuel L. Madorsky

## Abstract

Samples of polydivinylbenzene (PDVB) and of copolymers of styrene with divinylbenzene (DVB) and with trivinylbenzene (TVB) were pyrolyzed in a vacuum in the temperature range of 346 to 450 °C. The volatile products were collected in two fractions: A heavy fraction volatile at the temperature of pyrolysis and a light fraction volatile at room temperature. Mass spectrometer analysis of the light fraction showed that for the copolymer containing 2 percent DVB the yield of styrene monomer is somewhat greater than for the pure polystyrene. On pyrolysis, of copolymers containing 25 percent of DVB or of TVB yield reduced amounts of styrene monomer; those containing about 50 percent of DVB do not yield any styrene monomer. Rates of thermal degradation of PDVB and of the copolymers were studied in the temperature range of 330 to 390 °C; the activation energies calculated on the basis of these rates were 53, 54, 58, 58, 61, and 65 kcal/mole for the copolymers containing 2 percent DVB, 25 percent DVB, 48 percent DVB, 56 percent DVB, 25 percent TVB, and for PDVB, respectively.

## 1. Introduction

Polystyrene was found to decompose thermally in a vacuum [1]^2^ or in a neutral atmosphere [2, 3] at temperatures below 500 °C by a mechanism consisting of random scissions of C—C bonds, followed by unzipping at the resulting free-radical chain ends to yield monomers and multiples of structural units of an average molecular weight of about 264. Under these conditions complete volatilization of the sample takes place, for example, at 400 °C in about 30 min. of heating. On the other hand, polytrivinylbenzene (PTVB) [3], when pyrolyzed under similar conditions, does not yield any appreciable amounts of either styrene or of trivinylbenzene monomers. instead, the volatile degradation products consist of a fraction containing a small amount of hydrogen and saturated and unsaturated hydrocarbon fragments having from 1 to 11 carbons; a less volatile fraction consists of larger fragments having an average molecular weight of 372; and the residue consists of a carbonaceous material.

The mechanism of degradation of this highly cross linked PTVB polymer is assumed to be as follows: When a C—C bond in the short connecting links between the phenyl groups breaks, two reactions are possible: (a) A transfer of hydrogen may take place at the site of break so that one end becomes saturated and the other unsaturated, and (b) the two ends become free radicals. In the latter case the free radicals do not unzip to form monomers because of the cross linkages that hold the phenyl groups at other points. Instead, the free radicals abstract hydrogen from the surrounding medium and become saturated. As the number of such scissions increases, fragments of various sizes, which may or may not contain phenyl groups, split off from the chain, abstract some hydrogen from the residue, and vaporize. The residue in the meantime becomes carbonized in the process through loss of hydrogen.

Intermediate between these two extremes are copolymers of styrene with divinylbenzene (DVB) or with trivinylbenzene (TVB). An investigation of the thermal behavior of such copolymers was carried out by Winslow and Matreyek [4] in nitrogen at atmospheric pressure, at temperatures up to about 550 °C. They were interested primarily in the relative thermal stability of these copolymers and in the nature of the residues, and therefore did not study the volatile productions of degradation.

The present study was undertaken to obtain information on the mechanism of thermal degradation of these copolymers through analysis of the volatile products of degradation. A study was also made of the degradation of polydivinylbenzene (PDVB). The present work, as in the case of most of our other work on thermal degradation of polymers, was carried out in a vacuum. In addition to investigation of the nature of the volatile degradation products, the rates and activation energies of degradation were also determined. Allowing for the differences in the experimental conditions, our results are in fairly good agreement with those of Winslow and Matreyek.

## 2. Materials

The copolymers^3^ consisted of polystyrene containing the following amounts, in weight percent, of DVB or TVB: 2 percent DVB, 25 percent DVB, 48 percent DVB, 56 percent DVB, and 25 percent TVB. The PDVB was prepared by heating a pure grade of DVB monomer in an evacuated and sealed 6-mm Pyrex tube in an oven at 80 to 90 °C for 6 weeks. The tube was then opened, and the polymer, which was in the form of a rod, was heated further for 1 hr in an evacuated chamber at 120 °C. A loss of 8 percent, presumably monomer, took place during this heating step, but no additional loss occurred on further heating at this temperature.

## 3. Experimental Procedures

### 3.1. Pyrolysis

The experimental procedure used in the pyrolysis of PDVB and of the copolymers was the same as that used earlier in the pyrolysis of PTVB and other polymers [3]. Apparatus No. II described in reference [3] was used. Samples weighing 15 to 30 mg were heated in a vacuum by quickly moving a preheated furnace into position surrounding the sample for pyrolysis. Duration of heating was a 5-min period to heat up the sample from room temperature to the temperature of pyrolysis, followed by a 30-min period at the pyrolysis temperature. Fluctuation of the final temperature was ±2 °C. The residues were weighed, and the volatile products were collected and fractionated. The following volatilized products were obtained: (a) A waxlike fraction designated as V_pyr_, volatile at the temperature of pyrolysis, but not at room temperature, which consisted of large molecular fragments deposited in the apparatus just beyond the hot zone; (b) a light fraction, V_25_, volatile at room temperature, collected in liquid-nitrogen-cooled trap; and (c) a gaseous fraction, V_−190_, not condensable at the temperature of liquid nitrogen.

Fractions V_pyr_ and V_25_ were weighed, and the amount of V_−190_ was determined from pressure, volume, and chemical composition data. Fractions V_25_ and V_−190_ were analyzed in the mass spectrometer. Fraction V_−190_ was very small, less than about 0.1 percent by weight, and was found to consist primarily of CO. This is similar to the results obtained previously [1] in the case of polystyrene.

**Figure 1:**
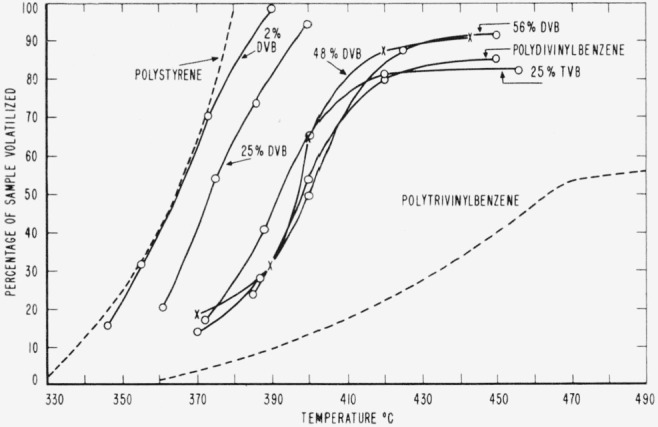
Relative thermal stability of polydivinylbenzene and polytrivinylbenzene, and their copolymers with styrene.

The relative thermal stabilities of PDVB and of copolymers of styrene with DVB and with TVB are shown in figure 1. Curves for polystyrene and PTVB, included in this figure for comparison, are based on data obtained previously [3, 5]. As can be seen from this figure, a styrene-DVB copolymer containing only a small percentage of DVB has a thermal stability not much different from that of the styrene homopolymer. The copolymer containing 25 percent of DVB shows an increase in stability over that of polystyrene. Still greater quantities of DVB in the copolymers produce further increases in the thermal stability, but at about 50 percent of DVB, stability reaches a maximum and is about equal to that of PDVB homopolymer. Polytrivinylbenzene has a much higher thermal stability than the PDVB, and it requires only 25 percent of TVB in the styrene-TVB copolymer to equal the thermal stability of PDVB.

The amounts of volatilized products from the pyrolysis experiments on PDVB and the copolymers are given in table 1. The V_25_ fractions from a number of experiments on the copolymers were analyzed in the mass spectrometer, and the results are shown in table 2. The mass spectrograph for fraction V_25_ from PDVB was very complex and could not be interpreted completely. The results are therefore not indicated in the table. However, there was a definite indication of the presence of considerable amounts of toluene, benzene, styrene, and xylene. There was also a group of peaks in the mass-range of 112 to 118, corresponding to a compound C_9_H_10_.

In the 2 percent-DVB copolymer the yield of the styrene monomer, C_8_H_8_, is slightly greater than in the case of 100 percent polystyrene, which was previously found to be about 40 weight percent of the total volatilized part [1, 5]. The yield of monomer decreases with the increase of DVB content in the copolymer and disappears altogether when the content of DVB is up to about 50 percent. There is, however, considerable monomer content in the volatiles from the 25 percent-TVB copolymer.

### 3.2. Rates

Rates of thermal degradation of the styrene copolymers and of PDVB were measured in a vacuum by a gravimetric method, which makes use of a very sensitive tungsten spring balance having a sensitivity of about 570 micrograms per millimeter of displacement. The apparatus and experimental procedure have been described in detail in an earlier publication [6]. Samples were limited to about 5 mg and were heated in a platinum crucible.

**Figure 2:**
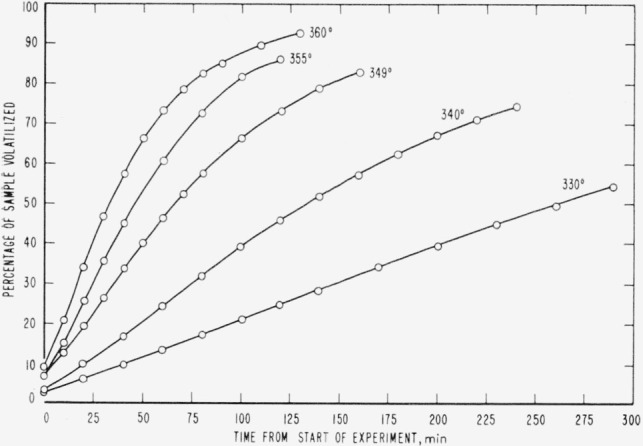
Thermal degradation of copolymer 98 percent styrene—2 percent divinylbenzene.

**Figure 3:**
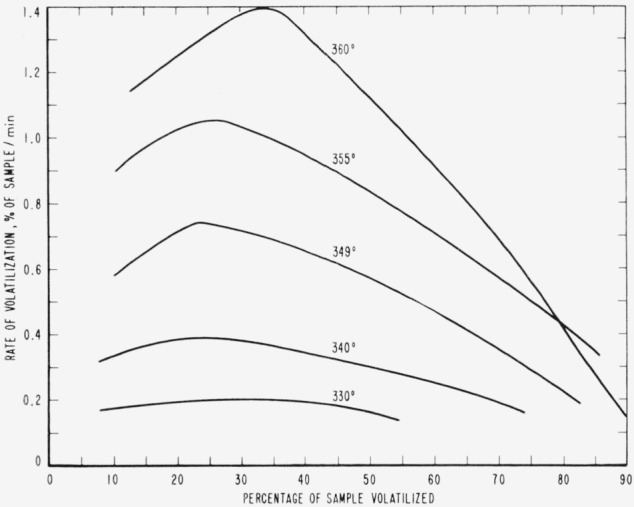
Rates of volatilization of copolymer, 98 percent styrene—2 percent divinylbenzene.

**Figure 4:**
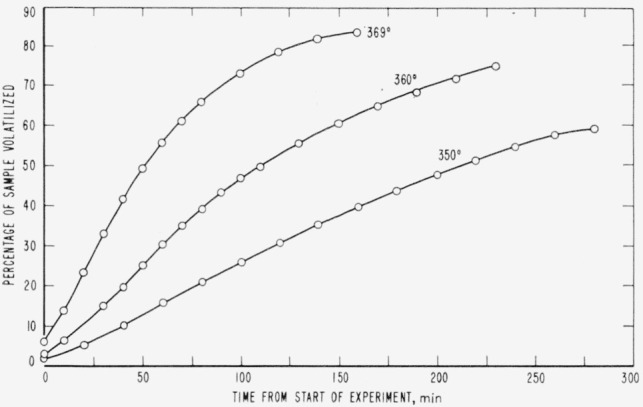
Thermal degradation of copolymer, 75 percent styrene—25 percent divinylbenzene.

**Figure 5:**
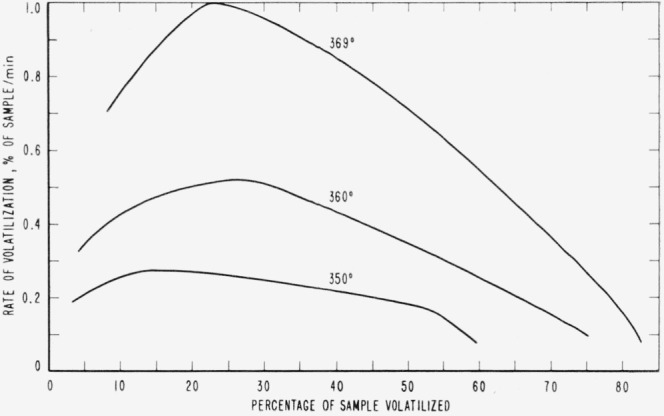
Rates of volatilization of copolymer, 75 percent styrene—25 percent divinylbenzene.

**Figure 6:**
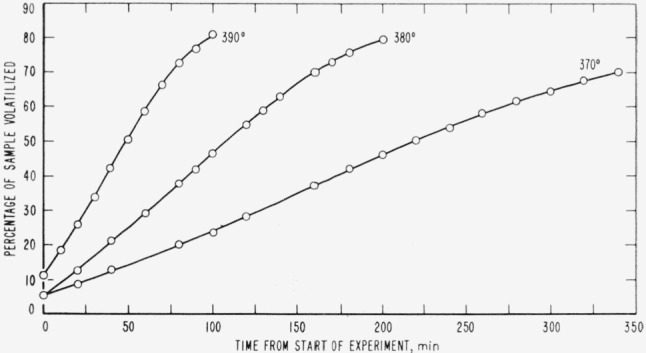
Thermal degradation of copolymer, 52 percent styrene—48 percent divinylbenzene.

**Figure 7:**
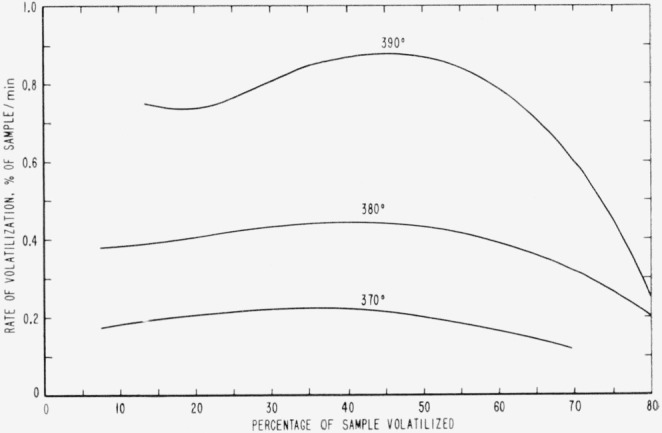
Rates of volatilization of copolymer, 52 percent styrene—48 percent divinylbenzene.

**Figure 8:**
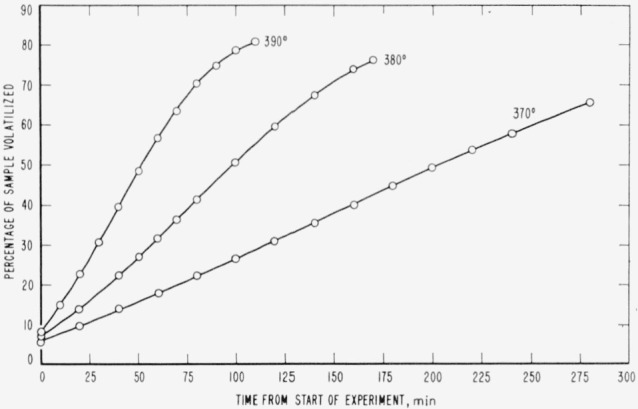
Thermal degradation of copolymer, 44 percent styrene—56 percent divinylbenzene.

**Figure 9:**
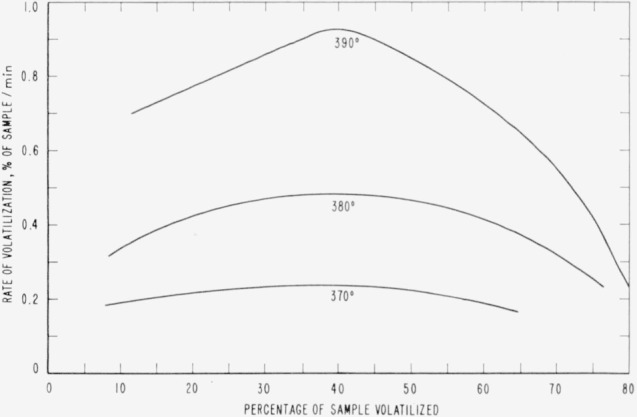
Rates of volatilization of copolymer, 44 percent styrene—56 percent divinylbenzene.

**Figure 10:**
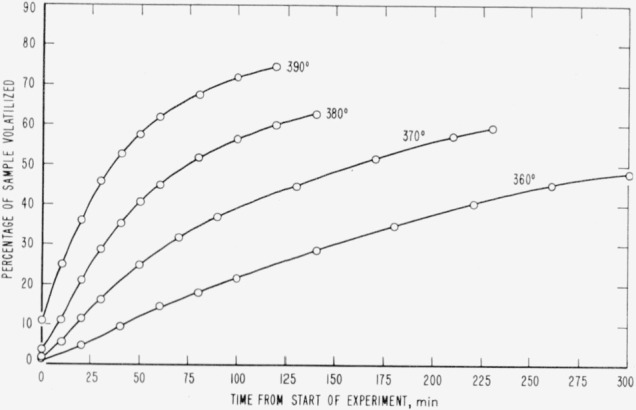
Thermal degradation of copolymer, 75 percent styrene—25 percent trivinylbenzene.

**Figure 11:**
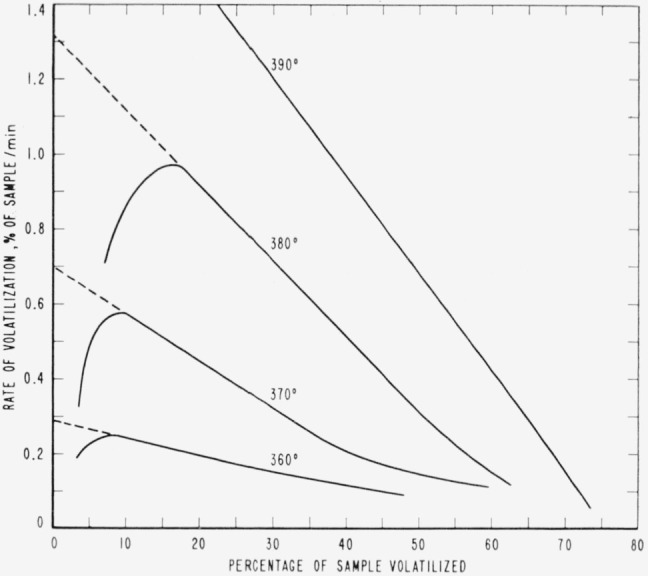
Rates of volatilization of copolymer, 75 percent styrene—25 percent trivinylbenzene.

**Figure 12:**
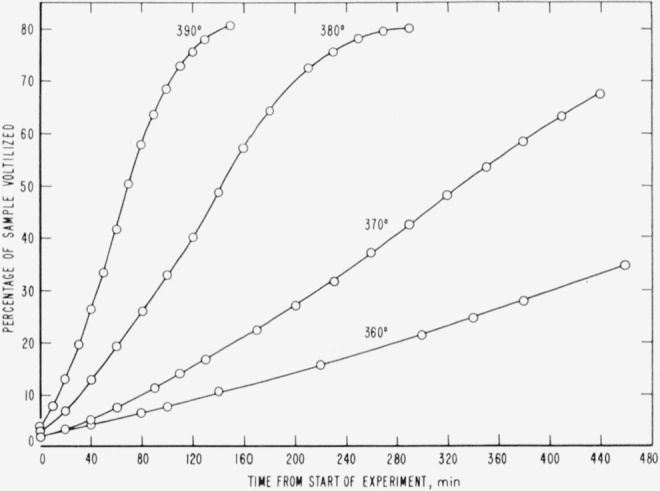
Thermal degradation of polydivinylbenzene.

**Figure 13:**
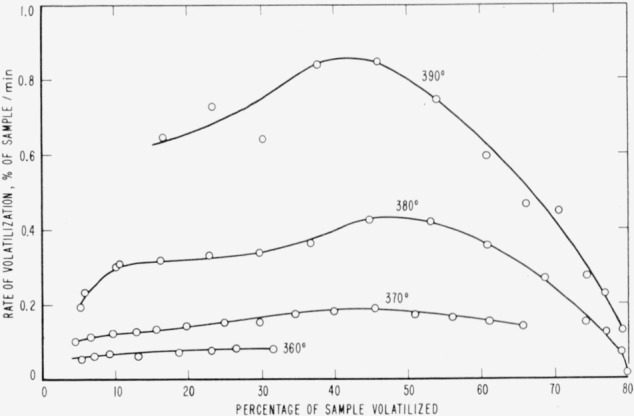
Rates of volatilization of polydivinylbenzene.

**Figure 14:**
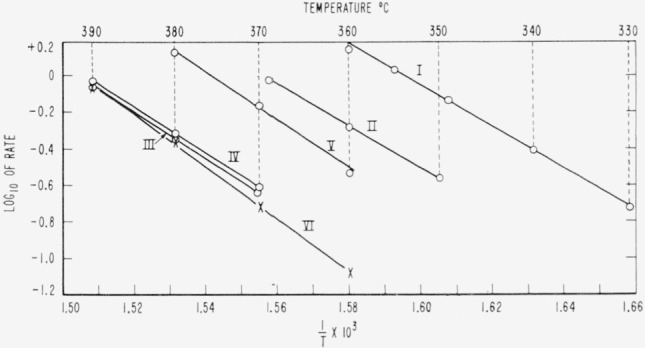
Activation energy curves for the thermal degradation of polydivinylbenzene and of copolymers of styrene with divinylbenzene and with trivinylbenzene: I, styrene—2 percent DVB, II, styrene—25 percent DVB, III, styrene—48 percent DVB, IV, styrene—56 percent DVB, V, styrene—25 percent TVB, VI, polydivinylbenzene.

Cumulative percentages of volatilization for the copolymers and for PDVB are shown plotted in figures 2, 4, 6, 8, 10, and 12. These curves of percentage loss versus time were used in calculating the data for the rate curves, which are shown plotted in terms of percentage loss per minute as a function of cumulative percentage volatilization in figures 3, 5, 7, 9, 11, and 13.^4^ In calculating the activation energies of the polymer degradation reactions the maximum rates were used in all cases except for the 25 percent TVB copolymer, where the initial rates, obtained by extrapolation (figure 11) were used. These maximums and initial rates, which are given in table 3, were used to prepare the curves in figure 14 and to calculate the activation energies of the polymers on the basis of the Arrhenius equation.

Numerical values of the activation energies based on the slopes of the straight lines in figure 14 are given in the last column of table 3. Data for PTVB [3] are shown for comparison. The activation energies increase with increase of cross linking agent in the copolymer. An activation energy for polystyrene was previously found to be 55 kcal/mole [7] which is about the same as for the copolymers having small percentages of DVB.

Additional calculations of activation energies for copolymers containing 2, 25, 48, and 56 percent DVB, were made on the basis of rates at 35 and 50 cumulative volatilization losses. The results of these calculations are shown in table 4. These results do not differ much from those based on the corresponding maximums (table 3).

## 4. Discussion

Allowing for the fact that in the work of Winslow and Matreyek [4] the degradation of the copolymers was carried out in nitrogen at atmospheric pressure, whereas a vacuum was used in the present work, the results are in close agreement, although our rates of degradation are a little higher, as expected.

The mechanism of thermal degradation of the copolymers of styrene with divinylbenzene or with trivinylbenzene can be visualized as follows: In polystyrene, cleavage of the chain ordinarily takes place at the weakest bonds, which are the C—C bonds in *β*-position to the double bonds in the phenyl groups—in this case the ones in the main chain.

**Figure:**
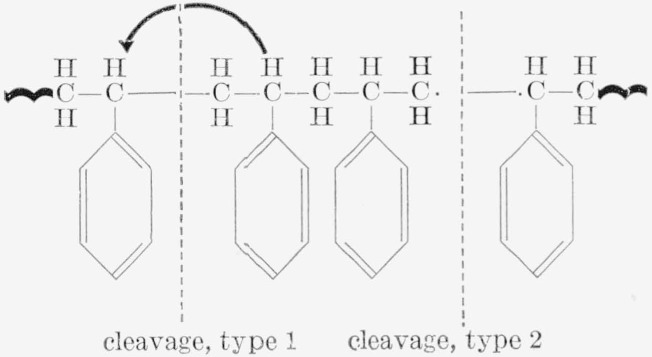


The cleavage can be of two types. Type 1 results in the formation of one saturated and one unsaturated end; and type 2 results in 2 free radicals, which proceed to unzip to give monomer, dimer, and trimer [1]. The monomer appears together with other small fragments in fraction V_25_, while the dimer, trimer, etc., appear in fraction V_pyr_. On the addition of a small amount of a crosslinking agent, such as 2 percent divinylbenzene, the unzipping to give dimer and trimer is somewhat blocked by the shortening of the free chain between cross links, and more of the monomer appears at the expense of the multiple units. However, with a further increase of cross links in the chain by the addition of DVB or TVB, the formation of monomer units is blocked.

The yield of monomer falls off for the copolymer containing 25 percent DVB, and even more for the copolymer containing 25 percent TVB. When the amount of the cross linking agent is still further increased, as in the case of copolymers containing 50 percent or more of DVB, no monomer appears among the volatile products.

There were no signs of carbonization of the degradation residues from copolymers with 2 or 25 percent of DVB. However, in the case of the copolymers containing higher percentages of DVB, 25 percent TVB, or of polydivinylbenzene, the volatilization curves begin to level off at about 80 to 90 percent loss (fig. 1), and carbonization of the residue takes place. The polytrivinylbenzened volatilization curve begins to level off at about 53 percent loss, and the residue shows considerable carbonization.

## Figures and Tables

**Table 1:** Pyrolysis of polydivinylbenzene and of copolymers of styrene with divinylbenzene and with trivinylbenzene^a^

Material	Temperature	Volatilization	Fractions, based on volatilization	
			V_pyr_	V_25_

	*°C*	%	%	%
I 2% DVB	$\{\;\begin{array}{c} 346\\ 355\\ 373\\ 390 \end{array}$	16.0 32.2 70.6 98.3	42.1 42.7 47.2 48.1	57.9 57.3 52.8 51.9
II 25% DVB	$\{\;\begin{array}{c} 361\\ 375\\ 386\\ 400 \end{array}$	20.7 54.2 73.9 94.5	43.0 49.7 49.1 52.1	57.0 50.3 50.9 47.9
III 48% DVB	$\{\;\begin{array}{l} 370\\ 390\\ 401\\ 420\\ 443 \end{array}$	18.6 31.1 64.6 87.5 90.7	80.5 87.4 94.9 96.0 95.0	19.5 12.6 5.1 4.0 5.0
IV 56% DVB	$\{\;\begin{array}{l} 370\\ 387\\ 400\\ 425\\ 450 \end{array}$	14.4 28.4 50.1 87.3 91.6	64.0 77.4 82.9 93.2 91.8	36.0 22.6 17.1 6.8 8.2
V 25% TVB	$\{\;\begin{array}{l} 372\\ 388\\ 400\\ 420\\ 450 \end{array}$	17.3 41.1 65.6 81.4 82.0	69.0 63.6 77.8 79.9 83.7	31.0 36.4 22.2 20.1 16.3
VI Polydivinylbenzene	$\{\;\begin{array}{c} 385\\ 400\\ 420\\ 450 \end{array}$	24.0 53.8 79.8 84.7	54.4 67.1 71.1 81.0	45.6 32.9 28.9 19.0

a Duration of heating in each experiment was 30 min, preceded by 5 min o preheating.

**Table 2:** Mass spectrometer analysis of volatile products from pyrolysis of copolymers of styrene with divinylbenzene and trivinylbenzene

Copolymer	2% DVB		25% DVB		48% DVB			56% DVB		25% TVB	

Temperature, °C	346	390	386	400	370	390	443	400	450	400	420

*Component*	*wt.* %	*wt.* %	*wt.* %	*wt.* %	*wt.* %	*wt.* %	*wt.* %	*wt.* %	*wt.* %	*wt.* %	*wt.* %
C_3_H_6_	……….	……….	0.8	……….	……….	……….	……….	……….	……….	0.8	0.4
C_4_H_6_	……….	……….	0.6	……….	……….	……….	……….	……….	……….	……….	……….
C_5_H_10_	……….	……….	……….	……….	……….	……….	……….	0.7	0.6	……….	……….
C_5_H_12_	……….	……….	……….	……….	……….	……….	……….	3.0	2.7	……….	……….
C_6_H_6_	……….	0.4	1.5	0.5	……….	……….	……….	……….	……….	0.4	0.4
C_6_H_14_	……….	……….	0.8	……….	……….	……….	……….	……….	……….	……….	……….
C_7_H_8_	0.6	1.4	2.8	2.7	……….	……….	……….	……….	……….	2.7	3.0
C_8_H_8_	51.7	49.8	39.9	34.8	……….	……….	……….	……….	……….	16.2	14.4
C_8_H_10_	2.0	……….	0.9	1.4	……….	0.5	0.6	1.0	1.2	1.3	1.4
C_9_H_10_	0.7	……….	1.7	1.0	0.4	0.4	0.5	0.9	……….	0.4	……….
C_9_H_12_	2.1	……….	0.7	0.8	0.7	0.8	0.6	0.9	0.9	……….	……….
C_10_H_12_	……….	……….	……….	4.8	9.2	7.6	2.0	6.7	0.5	……….	……….
C_10_H_14_	0.8	……….	1.0	1.1	8.9	2.9	0.6	1.7	0.5	……….	……….
Others^a^	0	0.3	0.2	0.8	0.3	0.4	0.7	2.2	1.8	0.4	0.5
	
Total of V_25_	57.9	51.9	50.9	47.9	19.5	12.6	5.0	17.1	8.2	22.2	20.1
V_pyr_	42.1	48.1	49.1	52.1	80.5	87.4	95.0	82.9	91.8	77.8	79.9
	
Total	100.0	100.0	100.0	100.0	100.0	100.0	100.0	100.0	100.0	100.0	100.0

a Components in amounts of 0.3 percent or less are not shown individually in this table.

**Table 3:** Rates of thermal degradation of polymers of divinylbenzene and trivinylbenzene and of their copolymers with styrene

Material	Temperature	Rate	Activation energy^a^

	°*C*	%/*min*	*kcal*/*mole*
I 2% DVB	$\{\;\begin{array}{l} 330\\ 340\\ 349\\ 355\\ 360 \end{array}$	0.19 .39 .74 1.05 1.39	} 53
II 25% DVB	$\{\;\begin{array}{c} 350\\ 360\\ 369 \end{array}$	0.27 0.52 1.00	} 54
III 48% DVB	$\{\;\begin{array}{c} 370\\ 380\\ 390 \end{array}$	0.23 .45 .88	} 58
IV 56% DVB	$\{\;\begin{array}{c} 370\\ 380\\ 390 \end{array}$	.24 .48 .93	} 58
V 25% TVB	$\{\;\begin{array}{c} 360\\ 370\\ 380\\ 390 \end{array}$	.29 .70 1.32 (b)	} 61
VI Polydivinylbenzene	$\{\;\begin{array}{c} 360\\ 370\\ 380\\ 390 \end{array}$	0.08 .19 .44 .86	} 65
VII Polytrivinylbenzene	$\{\;\begin{array}{c} 394\\ 420\\ 430\\ 440 \end{array}$	.03 .28 .59 1.22	} 73

a Activation energies for copolymers I, II, III, IV, and for homopolymer VI were calculated on the basis of maximum rates; those for copolymer V and for homopolymer VII, on the basis of initial rates.

b Degradation rate at this temperature was too fast to obtain an accurate extrapolated initial rate.

**Table 4:** Activation energies based on rates of degradation at 35 percent and 50 percent volatilization

DVB in copolymer	Percentage degradation	Activation energy

%	%	*kcal*/*mole*
I 2	$\{\;\begin{array}{c} 35\\ 50 \end{array}$	50 50
II 25	$\{\;\begin{array}{c} 35\\ 50 \end{array}$	56 56
III 48^a^	50	63
IV 56^a^	50	58

a In the case of copolymers containing 48 percent and 56 percent DVB, the maximums in figures 7 and 9, respectively, appear at percentages of volatilization higher than 35; therefore the rates at 35 percent volatilization were not used in calculating activation energies.

## Footnotes

2 Figures in brackets indicate the literature references at the end of this paper.

3 The authors are indebted to F. H. Winslow of Bell Telephone Laboratories for supplying them with samples of these copolymers.

4 In figure 13 the separate points are shown. The calculated rates at 390 °C are rather high, and the calculated points show a good deal of scattering.
